# Classifying the Perception of Difficult Life Tasks: Machine Learning and/or Modeling of Logical Processes

**DOI:** 10.11621/pir.2024.0205

**Published:** 2024-06-15

**Authors:** Ekaterina V Biyutskaya, Elyar E. Gasanov, Kseniia V. Khazova, Nikita A. Patrashkin

**Affiliations:** a *Lomonosov Moscow State University, Russia*

**Keywords:** situation perception, diffcult life task, orientation in a difficult situation, coping, machine learning, modeling of logical processes, complex diagnostics, coping classification, decision tree

## Abstract

**Background.:**

Although quite a few classifications of coping strategies have been proposed, with different premises, much less is known about the methods of interpretation and how people using different types of coping perceive their life difficulties.

**Objective.:**

To develop a verifiable algorithm for classifying perceived difficulties. The proposed classification was developed deductively, using “approach–avoidance” as the basis for cognitive activity aimed at taking on (approaching) a difficult situation or escaping from it, avoiding a solution to the problem. The classification comprises 1) driven, 2) maximal, 3) optimal, 4) ambivalent, and 5) evasive types of perception of difficult life tasks (DLTs). Types 1, 2, and 3 correspond to approaching a difficult situation, and 5 to avoiding it. Type 4 involves a combination of approach and avoidance.

**Design.:**

The type is determined by an expert psychologist in a complex way, based on a combination of 1) the respondent’s profile according to the “Types of Orientations in Difficult Situations” questionnaire (TODS) and 2) features that are significant for the type as shown in qualitative data — descriptions of DLTs (answers to open questions). Machine learning methods and A.S. Podkolzin’s computer modeling of logical processes are used to develop the algorithm. The sample comprised 611 adult participants (M_age_ = 25; SD = 5.8; 427 women).

**Results.:**

Using machine-learning algorithms, various options were tested for separation into classes; the best results were obtained with a combination of markup and questionnaire features and sequential separation of classes. Using computer modeling of logical processes, classification rules were tested, based on the psychologist’s description of the features of the type of perception. The classification accuracy using these rules of the final algorithm is 77.17% of cases.

**Conclusion.:**

An algorithm was obtained that allows step-by-step tracing of the process by which a classification problem is solved by the psychologist. We propose a new model for studying situational perception using a mixed research design and computer-modeling methods.

## Introduction

The development of typologies for the psychology of coping is a significant trend in current research. Such typologies are important because they allow us to generalize the different ways that people interact with stressful (and difficult) situations, as well as to develop evidence-based recommendations for psychological care. In this paper, we present a typology that is based on quantitative and qualitative data and allows us to describe people’s conceptions of coping in the structure of a perceived situation.

### Classifications in the Psychology of Coping

Considering the development of views on types of coping, we can identify various approaches to classifications and their justification. In earlier studies, the main question involved a search for the structure of coping. That is, solutions were found to the tasks of describing a) the features that make it possible to systematize lists of coping strategies, and b) levels of the coping structure (Pearlin & Schooler, 1978; Skinner et al., 2003).

Among the best-known premises used in the deductive approach to classification are *the functions of coping* in the adaptation process, which allow us to distinguish between problem-oriented and emotion-oriented variants of coping (Lazarus & Folkman, 1984); and a focus on approaching a stressful situation or avoiding it. This dimension (approach–avoidance) — “topological features” (Skinner et al., 2003, p. 225) — originates from work on exploratory behavior (Barnett, 1958). Later, those features began to be used to denote cognitive and emotional activity that is either focused on the perception of a stressor or on diverting attention from it (Roth & Cohen, 1986). By correlating approach–avoidance and coping strategies, the authors distinguish between ways of, on the one hand, facilitating contact with a stressful situation and, on the other, avoiding the problem. These types of behavior are not mutually exclusive, but can complement each other (Skinner et al., 2003).

The inductive way of grouping and structuring coping strategies is associated with the use of content analysis for qualitative data (for example, Daglas et al., 2024) and statistical procedures for processing quantitative data (exploratory and confirmatory factor analysis). One of the most recent trends proposes identifying types of coping by analyzing not the coping strategies themselves, but their combination or the profile which is determined from questionnaires using latent profile analysis and cluster analysis (Doron et al., 2015; Kavčič et al.; 2022; Muniandy et al., 2022; Nagy & Balázs, 2023). The basic idea is to measure and highlight typical coping patterns that appear in human behavior under stressful conditions. The profile is the combination of coping strategies revealed by the questionnaire. Such studies are most often performed in the context of a person-centered approach to coping, which means identifying *groups of people* with similar profiles, as opposed to the grouping of variables. This approach is also often based on the study of stable personality traits (Muniandy et al., 2022; Nagy & Balázs, 2023).

Thus, in the field of coping, quite a few options have been accumulated for solving the problem of classifying coping strategies. In some cases, classifications incorporate perceived characteristics of a situation (for example, perceptually controlling or changing the meaning of a problem as one coping reaction, in Pearlin & Schooler, 1978) or use correlations of the level of perceived stress with latent coping profiles or coping clusters (Chen et al., 2022; Doron et al., 2015; Muniandy et al., 2022). Nevertheless, the perception and interpretation of the difficult situation that the subject is coping with remain little-studied phenomena. Meanwhile, not only in the situational, but also in the person-oriented approach to the understanding of coping, there is a recognition of the leading role of “the interaction between an individual and the environment, involving subjective perception and assessment of stressors” (Lecic-Tosevski et al., 2011, p. 290).

### Conceptualization of an Image of the Difficult Life Situation

As E.A. Skinner and colleagues note, one of the most important features of the classifications of coping allows us to distinguish between different types of activity. Activity, which in this case is considered in the context of the German tradition as an “action schema,” is not identical to “behavior,” but also includes individuals’ emotions, attention, and goals. It is the goal and motivation that establish the directionality of behavior. The same coping behavior can reflect different types of activity if it is performed in the service of different goals (Brandstädter, 1998; Skinner et al., 2003). This approach is expressed in the following statement: “The structuring of coping modes as active behaviour patterns resulting from perception and cognitive processing is another arbitrary definition. Coping modes are in fact part of the overall coping process, but they constitute the behaviour patterns which can be actually observed, that is, which manifest themselves as the consequences of the entire process” (Heim, 1995, p. 147). Leontiev’s general psychological activity theory, which is close to this tradition, also postulates that activity is mediated by one’s image of the world (Leontiev, 1979).

In general, this approach makes it possible to consider coping as part of a more complex system — a perceived situation (or image of the situation), including sensory images, meanings, and personalized meanings regarding the event. Coping with a difficult life situation itself turns out to be a consequence of how this image functions (Asmolov et al., 2023). Using this approach in the present study allows us to study *the types* as *patterns of perception of difficult situations*. According to J. Rauthmann and R. Sherman, to the extent that there are individual differences in the perception of situations, people with similar levels or patterns of situation perception may be grouped together (Rauthmann & Sherman, 2019).

### Computer Modeling in Studies of the Psychology of Coping

Machine learning is used for various tasks in current studies on the psychology of coping: identifying predictors of stress (Tigga & Garg, 2022), studying behavioral patterns in response to stressors (Zhao et al., 2022), developing chatbots that teach coping skills (Fardouly, Crosby, & Sukunesan, 2022). At the time of writing, we were unable to find classifications of the perception of difficult or stressful situations, created using machine learning. ^1^ However, the topic is being actively developed in basic medicine. Models are being proposed that are designed to facilitate medical diagnostics and provide support for clinical decisions. Despite the recognition of the capabilities of data analysis using machine learning, it is noted that models are often based on a “black box” of decision making. Therefore, the need for interpretable models has been posed (Chen et al., 2021).

One of the approaches that makes it possible to achieve greater understanding of a model and to explain the path to a specific solution is Explainable AI (artificial intelligence). That is a general term for a wide range of computational instruments designed to improve understanding of the underlying mechanisms that drive predictions based on machine learning (C Manikis et al., 2023). Algorithms are used that create an interpretable model, for example, decision tree or logistic regression.

Another approach — the modeling of logical processes — is being developed by Russian mathematician A.S. Podkolzin. The author asserts that the central problem of artificial intelligence is the algorithmization of knowledge, and the main opportunity to create effective problem solvers is computer modeling of the logic of human reasoning (Podkolzin, 2008, p. 13). Let us note the fundamental difference between 1) Explainable AI and 2) the modeling of logical processes. The application of the first is associated with explanation of the proposed AI solution, as well as its comparison with expert opinion and common sense. The second approach is an explication of the human decision-making algorithm and modeling of this decision. We used modeling of logical processes to explicate the psychologist’s algorithm.

### The Present Study

**Figure 1.**
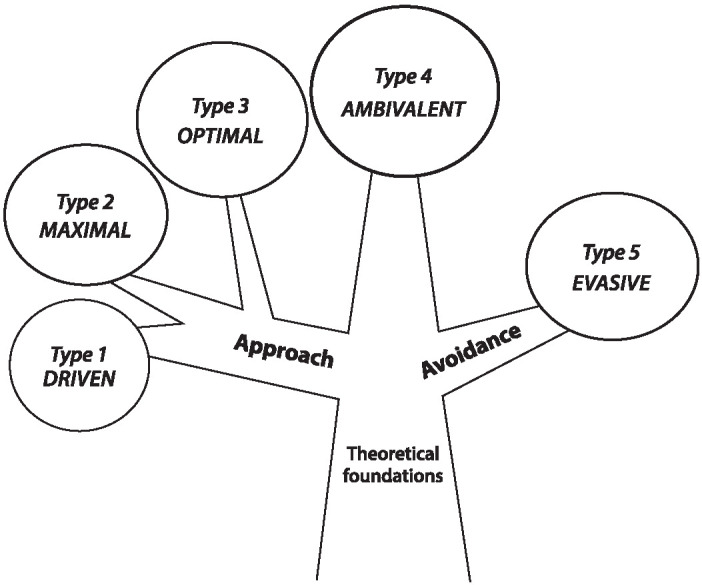
Types of perception of difficult life tasks

The classification of perceived difficulties that is presented in this work was developed based on information about difficult life tasks (DLTs). This is a type of difficult life situation involving an elevated and significant goal and the possibility of subjective control by the subject. The classification is based on the following theoretical premises: a conceptual model of types of orientation to a difficult situation (Bityutskaya, 2018), as well as the “approach–avoidance” dimension. While conducting the research and analyzing the empirical data, it turned out that the majority of respondents report simultaneously approaching and avoiding a difficult situation. Therefore, an ambivalent type was also identifi ed, which involves a combination of features of both approach and avoidance. Thus, our classification includes three major types of perception: approach, avoidance, and ambivalent perception. “Approach” is further divided into three subtypes: driven, maximal, and optimal (*Figure 1*).

*The purpose* of the study is to develop a verifiable, reproducible algorithm for identifying types of perceptions of DLTs.

For our study, it is the situational context that is important, not stable personality traits. We proceed from the assumption that a number of common characteristics that we have identified for groups of people allow us to describe similar patterns of perception of DLTs. We are studying coping in the structure of the subject’s image of the situation. This differs from studying coping strategies alone, and allows us to consider perception of and coping with a difficult life situation holistically. We consider the following components in the structure of the image of a subjective situation.

*Situational context* (time, place, life situation — for example, occupational difficulties, illness, etc.).

*Perception per se —* cognitive and emotional activity aimed at either perceiving/ approaching a difficult task or avoiding a solution to it (orientations), emotions, appraisal of its difficulty (including criteria and degree of difficulty, valence of appraisal).

*Objective —* what results need to be achieved in this situation.

*Coping —* how the objective is to be achieved.

*Conditions of the task —* help from the social environment, opportunities and limitations.

*Probable outcomes —* the best-case and worst-case scenarios for the situation.

## Methods

### Design

**Figure 2.**
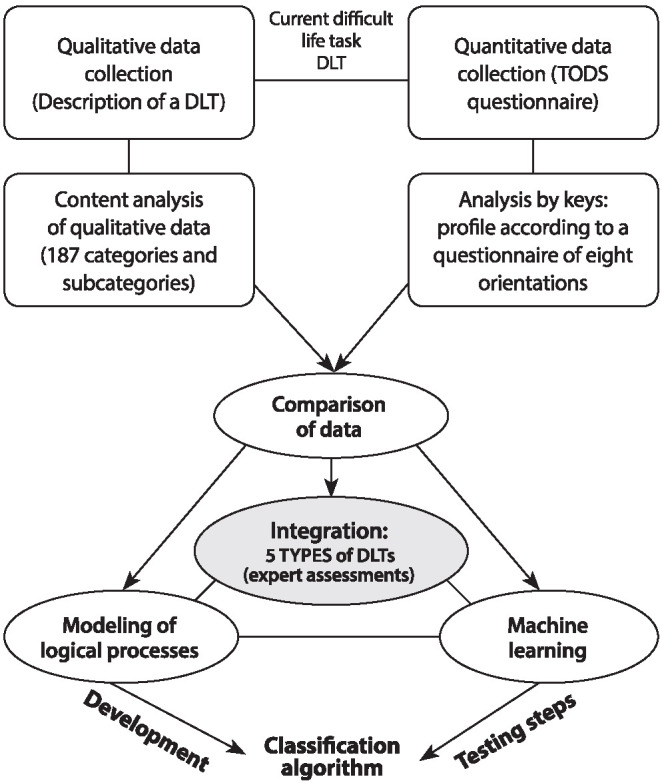
Mixed-methods research design

Study of the perception of a situation involves, on the one hand, consideration of a combination of different components, and on the other, analysis of individual parameters. The best solution seems to be a combination of quantitative and qualitative methods. Our study therefore adopted a mixed-methods research design using computer modeling. *Figure 2* demonstrates a convergent parallel research design. We collected quantitative and qualitative data simultaneously about one current DLT from each respondent. Initially, these data (the individual profile according to the questionnaire and the corresponding description of the DLT) were compared by a psychologist, who classified each case as one of the five types. The psychologist’s decisions were largely based on implicit knowledge. Then, using machine-learning methods, we tested different classification options, including some that the psychologist did not use. Based on A.S. Podkolzin’s approach, we developed a verifiable algorithm for assigning each description of DLTs to a certain type, explicating the psychologist’s solution to the problem in the process of algorithm development.

The need to use expert opinion at the beginning and computer modeling in the following stages arises due to the multidimensional nature of the data, the need to compare them and consider them holistically (187 qualitative data analysis units and 8 questionnaire scales are used). Data were integrated in two ways: through assessments by an expert psychologist and based on computer modeling.

### Study Participants and Material

The study involved 611 people, 184 men and 427 women (aged 19–52; M = 25; SD = 5.8), university students as well as working professionals with higher and secondary specialized education, residents of Moscow and Moscow Oblast. All respondents confirmed their voluntary participation in the study by giving informed consent. Each participant described one difficult life task that was relevant to them. The material provided for the study comprised various sorts of life difficulties: occupational, material, interpersonal, intrapersonal, and others.

### Data Collection

*The Structured Description of a Situation* includes introductory instructions about the formulation of a difficult life task and six open-ended questions about it (see Appendix). The method operationalizes the perception of difficult life situations and allows us to obtain qualitative data in the form of a *description of the DLT*. Each participant first described a relevant situation based on these questions, and then analyzed the same situation based on the questionnaire.

*The “Types of Orientations in Diffi cult Situation” questionnaire* (TODS; Bityutskaya & Korneev, 2020) was designed to diagnose how respondents perceive the difficult situation that they describe as relevant to them. The questionnaire comprises 65 items which respondents must answer relative to the situation described and assess on a Likert scale from 0 to 3 (0 — “absolutely wrong,” 1 — “somewhat wrong”; 2 — “somewhat right,” and 3 — “absolutely right”). The theoretical basis of the questionnaire provides a model of types of orientation (Bityutskaya, 2018). The model describes two types of cognitive and emotional activity: 1) approaching a difficult situation (focusing on it, to direct one’s efforts to change the situation), and 2) avoiding it (cognitive evasiveness, allowing one to ignore the difficulty and expend less effort). Based on the TODS, eight orientations can be identified. The first type pertains to the drive, thoroughness, and opportunity orientations; and the second type to rejection, inaction, and insouciance. Two scales — threat alert and obstacle orientation — can be combined with the orientations of both the first and the second types. When we tested the factor structure of the questionnaire, acceptable indicators of fit of the confirmatory model to empirical data were obtained: RMSEA = .049, CFI = .900, χ^2^(1171) = 3068.835.

### Data Analysis

The profile of the TODS study participants was considered comprehensively, as a *combination* of expressed orientations. Since orientations to threats and obstacles can apply to both approach and avoidance, we differentiated six scales: those related to approach (drive, thoroughness, opportunity orientation) and to avoidance (rejection, inaction, insouciance). At first, the types of perception of DLTs were identified based on the values on the scales in the individual profile, derived from the instruction scale (from 0 to 3 points), where 1.5 is the mean value. Accordingly, scores of 1.5 and higher were interpreted as expressions of an orientation.^2^

Content analysis (continuous counting) was used to process the qualitative data. The coding instructions for content analysis were developed by E.V. Bityutskaya and N.G. Malysheva using a bottom-up approach. Independent raters were used in development of the coding system, and consensus was reached on how to code unclear entries. The instructions include categories related to 1) description of the situation as a whole, and 2) individual issues. Thus, the unit of context was descriptions of situations (for the first type of categories) and answers to each question (for the second type of categories). The categories of the first type included emotions, time, energy, degree and essence of the difficulty. The categories of the second type were the nature of the situation, coping, several categories of appraisal, goals, opportunities, limitations, and others. The coding instruction includes 187 units of analysis — categories and subcategories. All 600 cases were independently coded by two coders, and the markings were then compared. Discrepancies were resolved through consensus. Content analysis was used for *the markup* from which the computer modeling was performed.

### Methods for Computer Classification of Difficult Life Tasks

We used two approaches to classify difficult life tasks:

1) Machine-learning algorithms (decision tree, logistic regression) for the purpose of modeling different classification variants;
2) A.S. Podkolzin’s modeling of logical processes in order to simulate the decision-making of a psychologist performing a classification task, and to optimize the procedure.

*Machine learning* was implemented in Python (Python Software Foundation) (Pedregosa, 2011). First, we applied the basic configurations of machine-learning algorithms — a set of default parameters in the scikit-learn library of the Python programming language. Then, each algorithm was tuned to improve classification accuracy.

*A decision tree* is a binary tree, each internal node of which is assigned a certain rule, which, according to the object to be classified, determines which of the branches to move on to. The decision tree is built from the training sample so as to classify the objects of the training sample as well as possible. If you choose a tree depth large enough, then, as a rule, it is possible to achieve 100% classification of the training sample; but this can lead to overfitting. Therefore, before constructing a decision tree, it is usually decided that its depth should not exceed a certain constant; in our work the depth should not exceed the number 4.

*Logistic regression* is a linear classifier for two classes — i.e., a classifier in which the surface separating two classes is a hyperplane in the feature space. The decisive rule for logistic regression is the following: all objects lying above the dividing hyperplane belong to the first class, and everything below belongs to the second. If the space of the features has a small dimension, then the coefficients describing the separating hyperplane can be interpreted and explained. Therefore, logistic regression can be considered to be Explainable AI.

We solved the problem of overfitting (the ability of machine-learning algorithms to adjust to the training sample so as to almost always give the correct answer for it) by using the cross-validation method. Our solution involves dividing the entire sample into training and testing parts in a ratio of 4:1, while accuracy is measured only on the testing part. This division was randomly performed 500 times, and the results were averaged.

*A.S. Podkolzin’s approach* involves the formulation and mathematical verification of simple, clear “decision rules” for the division into types. To establish classification accuracy, we measured the number of matches between *the expert’s assessments* of the case attribution and *the results of the decisive rule*. For example, an accuracy of 0.88 means that the rule can classify 88% of cases in a way that matches the expert’s opinion.

### Determining the Types of Perception of Difficult Life Tasks

The types were determined based on analysis of the indicators by an expert psychologist: 1) the respondent’s profile according to the TODS questionnaire, and 2) indicators significant for the type in the descriptions of the DLT.^3^ The latter are highlighted on the basis of a conceptual model of types of orientation in difficult situations (Bityutskaya, 2018); the following features were used:

*1 driven* (n = 67) — striving for difficulty associated with a feeling of drive. In the TODS respondent’s profile, the *drive* scale has the highest (or high) scores in combination with the expression of other orientations of approach to difficulties and a lack of expression of avoidance orientations. Qualitative data present indicators of positive assessments and emotions, self-development, increased energy, and high results.

*2 maximal* (n = 89) — multitasking and achieving a perfectionist goal with the greatest expenditure of effort. In the TODS profile, the *thoroughness* orientation is strongly expressed in combination with other orientations of approach to difficult situations and no expression of an avoidance orientation. The most significant features in the qualitative data are high achievement, a need to do everything, and multitasking*.*

*3 optimal* (n = 139) — focus on achieving a difficult goal with optimal efforts (no more, no less than required by the task conditions). In the TODS profile, orientations of approach to difficulties are expressed (with the highest indicator for *orientation towards opportunities*) and orientations of avoidance are not expressed. Frequent mention of planful problem solving and positive reappraisal of the situation, and goals expressing an approach orientation, are characteristic in the descriptions of DLTs.

*4 ambivalent* (n = 245)* —* fluctuation between approaching a difficulty and avoiding it (expressed by one, two or three orientations of approach in combination with one, two or three orientations of avoidance). The qualitative data show frequent mention of negative emotional states, which require time and effort to overcome; both the goals of approaching something pleasant and avoiding something unpleasant are described. Fluctuations in activity and passivity are possible when achieving a difficult goal.

*5 evasive* (n = 60) — avoidance of difficult emotional experiences that consume the consciousness, avoidance of a difficult situation (one, two or three scales of avoidance of difficulties are expressed, and scales of approach are not expressed). Intense negative emotions, coping, and avoidance goals are particularly common in the qualitative data*.*

A few descriptions of DLTs (n = 11) were not analyzed because they were not assigned to any of the types listed. The study was performed on a sample of 600 cases.

When the questionnaire profile and the indicators pointed to different types, a decision was made based on the questionnaire alone if the description was too brief (such cases accounted for no more than 1.5% of all descriptions). The remaining cases containing such a discrepancy were classified based on the DLT descriptions. There were also combinations in which both the questionnaire profile and the indicators equally pointed to two types — that is, the case turned out to be mixed. It was assigned to one of the two classes.

## Results

As a preliminary step, descriptive statistics were analyzed for a subsample of each type and for the entire sample (*Appendix*, *Table A1*). The expected results were obtained, according to which the most clear-cut scores are for those orientations that act as indicators of types. Orientations to threat signals and to obstacles have the smallest range of mean values (from 1.83 to 2.21 and from 1.53 to 1.89, respectively).

### Classification of Difficult Life Tasks Based on Machine-Learning Algorithms

#### Results of the Basic Configuration of the Algorithms

*Table 1* presents the results of the first stage — applying the basic configurations of the algorithms separately to two training samples: by markup (187 features; 1m) and by the questionnaire (8 features; 1q). In this way, one can see insufficiently high classification accuracy rates.

**Table 1:** Results of classification of DLTs based on machine-learning algorithms

Algorithm	Classification Accuracy		
	1m	1q	2k
Logistic regression	.445	.675	.712
Decision tree	.420	.658	.608

*Notes: 1m, 1q — the first stage, the results of applying the algorithms to the training set with a full set of features: 1m — according to markup, 1q — according to the questionnaire; 2k — the second stage, the results of applying the algorithms to a training set with 11 features: a combination of markup and questionnaire features.*

### Combination of Questionnaire Features and Markup

At the second stage, all the attributes we had were combined and an attempt was made to select the best ones for classification using the “sequential feature selection” method. This identified 11 features that provide the best accuracy: 6 TODS approach and avoidance scales and 5 markup features.

The results of applying the algorithms to the training set with 11 features are presented in *Table 1* (2k). Feature reduction and combination actions improved the classification accuracy score to **.712** (logistic regression).

### Sequential Separation of Classes

At the third stage, we tested an alternative variant, involving sequential (rather than simultaneous) separation of classes. To identify patterns in data distribution, we visualized the training sample of the questionnaire. *Figures 3a* and *3b* show images of the types in three-dimensional space, created on the basis of the indicators of the approach and avoidance scales of the TODS.

**Figure 3a.**
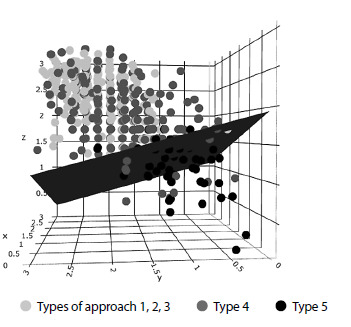
Visualization of types in three-dimensional space of TODS approach scales

**Figure 3b.**
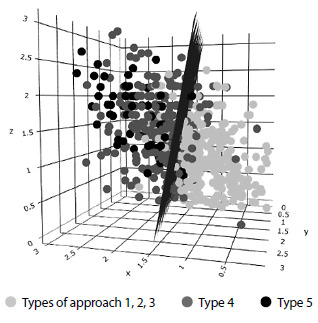
Visualization of types in three-dimensional space of TODS avoidance scales

In the figures we see that the type *5* can be linearly separated from the other types by features of approach (*Figure 3a*), and the three types of approach (*1, 2, 3*) by features of avoidance (*Figure 3b*). Therefore, in the first step, we try to divide the sample into three parts, first by separating type *5*, and second the types of approach. The third, remaining, part contains ambivalent cases. In the next steps it is necessary to separate the three types of approaching. In this case, first we separate types *1* and *2* from *3*, and then we separate *1* and *2*. Testing this model using the decision tree algorithm on a training set using the 11 features described above showed classification accuracy of **.783**. *Table 2* presents the results of the sequential class separation.

**Table 2:** Classification algorithm using machine learning (decision tree)

Step	Action	Classification accuracy at each step
1	We combine cases from the first three classes into one class (approaching) and divide the sample into 3 classes: 1,2,3 — 4 — 5	.895
2	We separate the 1st and 2nd classes from the 3rd: 1,2 — 3	.827
3	We separate classes 1 and 2: 1 — 2	.791
	Cumulative indicator	.783

Thus, in the machine-learning process, the best results were obtained by combining features and sequentially separating the classes. This division corresponds to the psychologist’s logic when determining the types, and we used it at the stage of modeling logical processes.

### Classification of Difficult Life Tasks Based on Computer Modeling of Logical Processes

At this stage, we test the accuracy of the classification using decision rules created based on the features of case categorization provided by the psychologist (see section “Determining the Types of Perception”).

### Development of a Classification Algorithm

In *the first step* of classification, based on the concepts of approach, avoidance, and ambivalent perception, the psychologist suggests using the following first rule:

- if in the TODS profile at least one of the approach orientations is expressed, and none of the avoidance orientations, then the case belongs to the types of approach (*1, 2, 3*);
- if at least one of the orientations of avoidance is expressed, and none of the orientations of approach, then the case belongs to type *5*;
- if at least one of the orientations of avoidance is expressed, and at least one of the orientations of approach, then the case belongs to type *4*;
- cases containing a profile in which no orientation is expressed are considered unclassifiable in this work.

Analysis of the training sample shows that this rule performs the classification correctly in **84.83%** of cases.

Next, to refine the orientation expression thresholds on the TODS, we perform a search of all possible sets of threshold boundaries of scales ranging from 1.0 to 2.1 in increments of 0.05. This analysis shows that if we consider *thoroughness* to be expressed when its corresponding number is greater than 1.65, and *insouciance* to be expressed when its corresponding number is greater than 1.85, while keeping the remaining thresholds equal to 1.5, then the rule described above in our sample produces correct classification in **88.5%** of cases. Therefore, in the first step we use the rule described above with refined expression thresholds.

In *the second step*, we separate the first two types related to approaching, from the third. In so doing, we proceed from the following features of the types. Types *1* and *2* are distinguished by the fact that the subject sets a high goal in a difficult situation; it involves expenditure of effort to a greater extent than the situation demands. Type *3* assumes that when achieving a difficult goal, as much effort is expended as required by the conditions of the task. Types *1* and *2* are identified by maximum expression in the TODS profile of the *drive* and *thoroughness* scales. In addition, analysis of the empirical data showed that the nature of situations corresponding to *1* and *2* is not characterized by a description of extreme situations: threats to life and health, illnesses, loss of loved ones (category F6 markup).

Based on what has been discussed above, we formulate the second rule, which is based on the TODS respondent’s profile vector, and also takes into account the mention of category F6:

- if in the description of the DLT there is no category F6 (*F6* = 0) and in the profile the maximum value among the approach scales is obtained on the “drive” and/or “thoroughness” scales, then we classify the case as belonging to the first two classes (driven and maximal types *1, 2*);
- otherwise we classify the case as type *3*.

The accuracy of separating approach types based on this rule is **76.92%** of cases.

It remained for us to separate the first two types (driven and maximal). In *the third step*, we use only the markup vector to solve this problem. The psychologist described 16 subcategories which characterize types *1* and *2* (we denote them “Dictionary 1” and “Dictionary 2,” respectively), and assigned a weight to each subcategory from 1 (least significant) to 3 (most significant). Lists of subcategories (Dictionaries 1 and 2) are given in the Appendix (*Table A2*). The *third rule* is formulated as follows:

- using the respondent’s markup vector, we calculate the weighted sums of the subcategories for each type;
- if the weighted sum of the subcategories of Dictionary 1 turns out to be greater than the weighted sum of the subcategories of Dictionary 2, then we classify the case as *1*;
- otherwise we classify it as type *2*.

The accuracy of separation of the first and second types, obtained at this stage by applying the third rule, is **88.04%** of cases.

**Figure 4.**
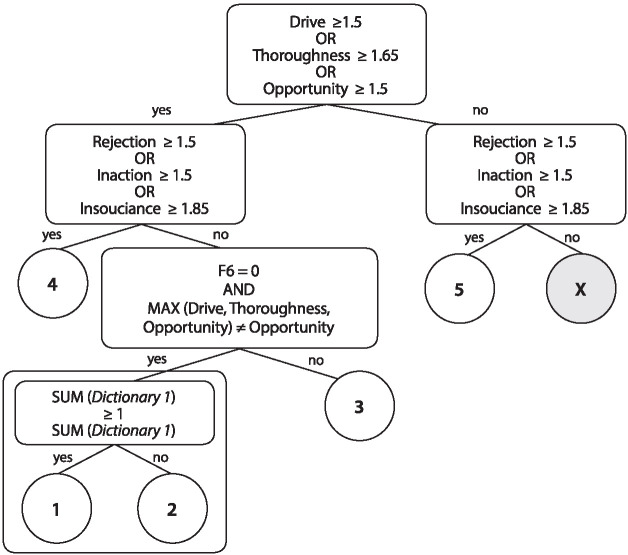
Decision tree of the final classification algorithm (modeling of logical processes)

Overall, on the available training set, this procedure correctly classifies **77.17%** of cases (cumulative indicator). The decision tree of the final algorithm is presented in *Figure 4. Table 3* shows the classification confusion matrix.

**Table 3:** Classification confusion matrix using the resulting algorithm

Classes	1st* class	2nd* class	3rd* class	4th* class	5th* class	Total	Correctly defined
*1*	38	10	10	9	0	67	56.7%
*2*	1	43	22	23	0	89	48.3%
*3*	7	18	98	16	0	139	70.5%
*4*	0	1	4	233	7	245	95.1%
*5*	0	0	0	9	51	60	85.0%

Note. * — the class to which the final algorithm assigned the cases; the number of matches to the expert’s assessment is highlighted by shading.

Analysis of the confusion matrix allows us to present two reasons for the inconsistency of cases with the rules of the final algorithm: 1) insignificant (.01–.03) excesses of the value of one scale relative to the threshold of expression; 2) the description of the DLT and the profile according to the questionnaire indicated diffierent types, while the psychologist, when deciding on assignment to a type, relied on the description; 3) a mixed type, which could have been attributed, among other things, to the type to which the algorithm assigned it. We see from the table that the largest number of “errors,” or cases that do not correspond to the final algorithm, belong to types of approach that are difficult to separate. The highest rate of agreement between expert and algorithm assessments was obtained for the ambivalent type.

## Discussion

In this study, we solved the task of developing a reproducible, verifiable algorithm for determining the types of perception of DLTs. Two computer modeling approaches were used: 1) machine learning and 2) logical process analysis.

The use of machine-learning algorithms made it possible to consider and evaluate the accuracy of different options for separating the data array into classes, and also to determine the optimal three-step path (*Table 2*).

In the context of Explainable AI, it is interesting to analyze the 11 features that were identified during the machine-learning process. Six of them are correctly defined by the TODS scales, which matched the opinion of the psychologist. Of particular interest are 5 features isolated from a set of 187 markup features. Analysis shows that this set includes the following features:

- 1D4 — subcategory “planful coping,” whose frequency of mention in the qualitative data allows us to distinguish types *1, 2, 3* from *4, 5*;
- Two features separating *1, 2* from *3* are C6 — a subcategory that describes the need to achieve maximum results, and F6 — the category “threat to life and health,” which is not found in descriptions corresponding to types 1, 2;
- Two features that uniquely characterize type *1* (A1 — subcategory “positive intense emotions”) and type *2* (1B9 — subcategory of “necessity”), which the psychologist also identified on the basis of content analysis and rated as highly significant for these types.

Overall, this decision seems logical and comprehensible. Note the absence of markup signs distinguishing type *5*. This is justified, because among all types it is distinguished with the greatest accuracy from the rest of the array, according to the TODS data.^4^

A.S. Podkolzin’s approach allowed us to test variants of rules based on the description of features by a psychologist and to work out a final decision-making algorithm. It turned out that at the first step of separation into three large types, the classification accuracy was quite high. However, the accuracy decreased in the second step, when dividing types *1, 2* –* 3*. There are two reasons for this: 1) similar semantic characteristics for the types of approach (manifested both in the similarity of profiles and semantic themes in the descriptions of DLTs); 2) mixed types (there are features of two types in one description).

Let us first analyze the semantic reasons. The first and second types, striving to achieve a goal that exceeds the requirements of the situation, tend to overestimate the efforts required, which is probably based on an overestimation of their own strengths and capabilities. In this case, it seems that we are dealing with positive illusions, which are associated with beliefs about the world and about oneself that are poorly supported by the facts, and form a more positive view on the part of the subject than is justified (Jefferson, Bortolotti, & Kuzmanovic, 2017). That is, one of the semantic reasons why it is difficult to separate the three types of approaching, relying only on a questionnaire, is if there are some illusory assessments of the situation and one’s capabilities in this situation, characteristic of people who perceive DLTs according to the driven and maximal types. The person *would thus like* to optimize their efforts, but *in reality* uses more effort than the situation requires. As a result, statements that relate to opportunity orientation can be rated as highly as items from the *drive* and *thoroughness* scales, as confirmed by analysis of the descriptive statistics (*Table A1*). At the same time, in accordance with our final algorithm, even with a slight advantage (by 0.01) of the score on the opportunity scale (compared to drive and thoroughness), the case should be classified as an optimal type.

The issue of *mixed types* requires separate analysis. Regarding psychological classifications, the idea of the rarity of “pure” types, sometimes the impossibility of identifying them in life (but only with a theoretical description), has become a truism. In our data, some cases were identified as a classification error by the final algorithm, precisely due to the *mixed type*. For such cases, as a rule, one can detect not only a discrepancy between the case and the rule, but also significant features of two types in the descriptions of DLTs. For example, quite often drive or maximum cases containing a description of emotional burnout and physical exhaustion (which characterizes ambivalent perception) were classified as ambivalent based on the first rule. Analysis of the data, including a detailed confusion matrix (with case numbers), showed that in our sample, at least 25% of the total number of respondents could be classified as mixed cases.

**Figure 5.**
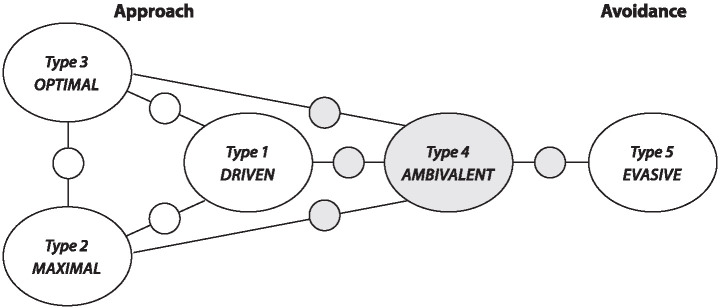
Visualization of the five main and mixed types in the form of a graph

*Note. The small circles indicate mixed subtypes, with the shading indicating the ambivalence of the subtype when mixed.*

This analysis allows us to propose a graph metaphor for visual representation of the typology **(***Figure 5***)**. Considering the significant number of cases of mixed types, this metaphor reflects more fully than a tree the idea of classification that we arrived at as a result of the study. However, this classification option requires further analysis and description. In particular, it remains to be determined whether specific traits can be described for mixed subtypes or whether they rather involve a combination of the two types of traits.

## Conclusion

This study uses a method for studying situational perception that involves a combination of 1) qualitative and 2) quantitative data. The first corresponds to the need to embrace the diversity of people’s individual conceptions. The second involves studying the profile derived from a questionnaire, which allows us to take into account the complex interaction of a person with a difficulty.

A final algorithm was developed that opens up the possibility of determining the type of perception of a difficulty, and not just individual parameters of the perceived situation. The approach to developing the algorithm used in this work, A.S. Podkolzin’s computer modeling of logical processes, makes it possible to optimize the classification of perceived difficult situations into certain types, and also to trace the process of a psychologist solving the classification problem step by step. This is a more meaningful classification option than using machine-learning methods. Its simplicity (including computational simplicity) and the greater ease of interpretation of the modeling of logical processes allow us to recommend this particular method for practical use. It involves taking into account scale thresholds and following the resulting algorithm.

In accordance with the results of the study, in order to divide the array of responses into three large types — 1) approach, 2) avoidance, 3) ambivalent perception of DLTs%— it is possible to implement a rule that uses the results of the questionnaire, that is, the part of the data that is the least labor-intensive to process. Approach includes three more subtypes; two of them are separated from the third also on the basis of the questionnaire, with the addition of one markup feature. Conducting a structured description of a situation involving the collection of qualitative data is necessary more to separate the first two types of approach (the driven and maximal types, to which 24% of the total sample in this study belonged).

## Limitations

The proposed classification is based on an analysis of the life difficulties of mostly young people living in a Russian metropolis who have or receive higher education. It is possible that analysis of other life contexts and age categories could highlight other types of perceived difficulties. There are also limitations to the methods. The reliability of conclusions based on machine learning could be increased by increasing the sample size. There are no such restrictions for modeling logical processes, but it would be important to check the classification accuracy using the final algorithm on a new data set. This is the perspective of this study.

## Footnotes

1 Search in databases: https://pubmed.ncbi.nlm.nih.gov/; https://psycnet.apa.org/; https://elibrary.ru/. Search queries on the topics “psychology”: perceived stress, coping classi! cation, machine learning, decision tree, logistic regression). Accessed: 2023.

2 The choice of this limit (rather than the sample average) is justified by the fact that it is important for us to correlate the respondent’s indicators with the frequency rating scale specified in the instructions, and not to compare these indicators with relative values for the sample.

3 In this case, knowledge about the type as a whole was used, in accordance with the conceptual model, regardless of the markup.

4 Additionally, we separated each class from all others using the logistic regression algorithm (the training sample of the questionnaire, 6 features). $ e best result was demonstrated by class 5%— accuracy 0.92.

## Appendix A

**Structured Description of a Situation**

Think of a situation in your life that involves a difficult task, requiring a solution in a given period of time:

1. How do you perceive it, evaluate it, experience it emotionally, and overcome it (what actions help you overcome the situation or your condition)?
2. What are your goals in this situation?
3. What opportunities and limitations do you have in achieving your goal?
4. Do you need help (support) from people around you in this situation?
5. If everything goes wrong, what will it be like? (Maximum failure).
6. Describe what would achieve the maximum success and would resolve the situation for you.

**Table A1:** Descriptive statistics of the TODS scales for types and the entire sample

Scales	Types					All
	*1*	*2*	*3*	*4*	*5*	
	Mean (SD)	Mean (SD)	Mean (SD)	Mean (SD)	Mean (SD)	Mean (SD)
Drive	2.46*** (.32)	2.00 (.52)	1.86 (.53)	1.49 (.53)	.89 (.39)	1.70 (.64)
Thoroughness	2.15 (.46)	2.46*** (.39)	2.07 (.48)	1.83 (.57)	1.11 (.40)	1.94 (.61)
Opportunity orientation	2.24 (.43)	2.32 (.41)	2.35*** (.38)	2.00 (.47)	1.21 (.33)	2.07 (.54)
Obstacle orientation	1.53 (.47)	1.77 (.49)	1.70 (.54)	1.89 (.57)	1.68 (.52)	1.77 (.55)
Threat alert	2.04 (.51)	2.21 (.52)	2.14 (.43)	2.05 (.54)	1.83 (.55)	2.07 (.52)
Rejection	.98 (.44)	1.22 (.41)	1.14 (.35)	1.90 (.42)	2.24*** (.42)	1.55 (.59)
Inaction	.65 (.46)	.74 (.43)	.83 (.37)	1.15 (.53)	1.43 (.54)	.99 (.53)
Insouciance	1.02 (.55)	.93 (.57)	.86 (.47)	1.39 (.62)	1.71 (.51)	1.19 (.63)

*Note. * — the most significant scale for determining this type, in accordance with the conceptual model; for type 4 there is no such scale, because in an individual profile classified as type 4, different scales of approach and avoidance can be expressed.*

**Table A2:** Subcategories characterizing types 1 and 2

Dictionary 1			Dictionary 2		
Category	Subcategory	Weight	Category	Subcategory	Weight
Energy	1. High energy level	3	Energy	1. Low energy level	1
	2. Energy is rising	3		2. Need for energy expenditure	1
Emotions	3. Positive intense	3	Time	3. Mentioned	2
	4. Positive non-intense	3		4. Temporary, transient situation	1
Valence of appraisal	5. Positive appraisal of the situation	3	Nature of the situation	5. Time allocation	3
	6. Ambivalent appraisal	1	Essence of the difficulty	6. To be able to do everything	3
Criteria of appraisal	7. Control of the situation	2		7. Need to succeed	3
Basis of appraisal	8. Dimensions	3		8. Need to achieve the maximum result	1
	9. Challenge	3	Basis of appraisal	9. Necessity	3
Coping	10. Positive reappraisal	2	Coping	10. “Struggle”	1
Goal	11. Development, expansion	3		11. To encourage oneself	1
Opportunities	12. It is noted that there are many opportunities	2	Goal	12. Preservation of what one has	1
	13. Self-development as an opportunity	3	Limitations	13. Work/study schedule limitations	1
Limitations	14. No limitations	2		14. A great deal to do and the inability to refuse multiple tasks	2
Prediction of failure	15. Impossibility of failure	2	Nature of failure	15. Postponement of the result to a later date	1
Nature of success	16. Fanciful success	1	Nature of success	16. To finish	1

## References

[c001] Asmolov, A.G., Bityutskaya, E.V., Bratus, B.S., Leontiev, D.A., Ushakov, D.V. (2023). Dialogues on/in the field of meanings: to the 120th anniversary of Alexey Nikolayevich Leontiev. Lomonosov Psychology Journal, 2(46), 5–22. 10.11621/LPJ-23-13

[c002] Barnett, S.A. (1958). Exploratory behavior. British Journal of Psychology, 49, 289–310. 10.1111/j.2044-8295.1958.tb00667.x 13596571

[c003] Bityutskaya, E.V. (2018). Tipy orientatsiy v trudnykh situatsiyakh [Types of orientation in difficult situations]. Voprosy Psikhologii, 5, 41–53.

[c004] Bityutskaya, E.V., & Korneev, A.A. (2020). Diagnostika vospriiatiia zhiznennykh trudnostei: situatsionnyi oprosnik “Tipy orientatsii v trudnoi situatsii” [Diagnostics of perception of life events: the situational version of the questionnaire “types of orientations in difficult situation”]. Vestnik Moskovskogo gosudarstvennogo oblastnogo universiteta (elektronnyi zhurnal) [Bulletin of Moscow Region State University (e-journal)], 4, 141–163. 10.18384/2224-0209-2020-4-1047

[c005] Brandtstädter, J. (1998). Action perspectives on human development. In Damon, W. & Lerner R. M. (Ed.), Handbook of child psychology: Vol. 1. Theoretical models of human development (pp. 807–863). New York: Wiley

[c006] Chen, B.W., Gong, W.J., Lai, A.Y., Sit, S.M., Ho, S.Y., Wang, M.P., &, Lam, T.H. (2022). Patterns of Perceived Harms and Benefits of the COVID-19 Outbreak in Hong Kong Adults: A Latent Profile Analysis. International Journal of Environmental Research and Public Health, 19(7):4352. 10.3390/ijerph19074352 35410033 PMC8998563

[c007] Chen, T., Shang, C., Su, P., Keravnou-Papailiou, E., Zhao, Y., Antoniou, G., ..., & Shen, Q. A. (2021). Decision Tree-Initialised Neuro-fuzzy Approach for Clinical Decision Support. Artificial Intelligence in Medicine, 111, 101986. 10.1016/j.artmed.2020.101986 33461686

[c008] C Manikis, G.C., Simos, N.J., Kourou, K., Kondylakis, H., Poikonen-Saksela, P., Mazzocco K., ..., & Fotiadis D. (2023). Personalized Risk Analysis to Improve the Psychological Resilience of Women Undergoing Treatment for Breast Cancer: Development of a Machine Learning–Driven Clinical Decision Support Tool. Journal of Medical Internet Research, 25, 43838. 10.2196/43838 PMC10337304 37307043

[c009] Daglas, Z., Lu, S., Gresham, D., Tatnell, R., Stanley, B.H., & Melvin, G.A. (2024). Classifying coping strategies from suicide prevention safety plans. Suicide and Life- Threatening Behavior. 10.1111/sltb.13039 38300145

[c010] Doron, J., Trouillet, R., Maneveau, A., Ninot, G., & Neveu, D. (2015). Coping profiles, perceived stress and health-related behaviors: a cluster analysis approach. Health Promot International, 30(1), 88–100. 10.1093/heapro/dau090 25324530

[c011] Fardouly, J., Crosby, R. D, & Sukunesan, S. (2022). Potential benefits and limitations of machine learning in the field of eating disorders: current research and future directions. Journal of Eating Disorders, 10(1), 66. 10.1186/s40337-022-00581-2 35527306 PMC9080128

[c012] Heim, E. (1995). Coping-based intervention strategies. Patient Education and Counseling, 26(1–3), 145–151. 10.1016/0738-3991(95)00733-g 7494714

[c013] Jefferson, A., Bortolotti, L., & Kuzmanovic, B. (2017). What is unrealistic optimism? Consciousness and Cognition, 50, 3–11. 10.1016/j.concog.2016.10.005 PMC5380125 27815016

[c014] Kavčič, T., Avsec, A., & Zager, K.G. (2022). Coping profiles and their association with psychological functioning: A latent profile analysis of coping strategies during the COVID-19 pandemic. Personality and Individual Differences, 185, 111287. 10.1016/j.paid.2021.111287 34584300 PMC8461263

[c015] Lecic-Tosevski, D., Vukovic, O., & Stepanovic, J. (2011). Stress and personality. Psychiatriki, 22(4), 290–297. 22271841

[c016] Leontiev, A.N. (1979). Psikhologiia obraza [Psychology of the image]. Vestnik Moskovskogo gosudarstvennogo universiteta. Seriia 14. Psikhologiia [Bulletin of Moscow State University. Psychology], 2, 3–13.

[c017] Muniandy, M., Richdale, A.L., & Lawson, L.P. (2022). Coping-resilience profiles and experiences of stress in autistic adults. Autism Research, 15(11), 2149–2166. 10.1002/aur.2817 36114687 PMC9826183

[c018] Nagy, L., & Balázs, K. (2023). Typical coping patterns: A person-centered approach to coping. New Ideas in Psychology, 70, 101023. 10.1016/j.newideapsych.2023.101023

[c019] Pedregosa, F., Varoquaux, G., Gramfort, A., Michel, V., Thirion, B., Grisel, O., ..., & Duchesnay, É. (2011). Scikit-learn: machine learning in Python. Journal of Machine Learning Research, 12(85), 2825–2830. 10.3389/fninf.2014.00014

[c020] Pearlin, L.I., & Schooler, C. (1978). The structure of coping. Journal of Health and Social Behavior, 19, 2–21. 649936

[c021] Podkolzin, A.S. (2008). Komp’iuternoe modelirovanie logicheskikh protsessov. Arkhitektura i iazyki reshatelia zadach [Computer simulation of logical processes. Architecture and languages of the problem solver]. M.: Fizmatlit.

[c022] Rauthmann, J., & Sherman, R. (2019). Toward a research agenda for the study of situation perceptions: A variance componential framework. Personality and Social Psychology Review, 23(3), 238–266. 10.1177/1088868318765600 29681223

[c023] Roth, S., & Cohen L. (1986). Approach, avoidance, and coping with stress. American Psychologist, 41(7), 813–819. 10.1037//0003-066x.41.7.813 3740641

[c024] Skinner, E.A., Edge, K., Altman, J., & Sherwood, H. (2003). Searching for the structure of coping: A review and critique of category systems for classifying ways of coping. Psychological Bulletin, 129(2), 216–269. 10.1037/0033-2909.129.2.216 12696840

[c025] Tigga, N.P., & Garg, S. (2022). Prediction of Global Psychological Stress and Coping Induced by the COVID-19 Outbreak: A Machine Learning Study. Alpha Psychiatry, 23(4), 193–202. 10.5152/alphapsychiatry.2022.21797 36425737 PMC9590681

[c026] Zhao, Y., Ding, Y., Shen, Y., Failing, S., & Hwang, J. (2022). Different coping patterns among us graduate and undergraduate students during COVID-19 pandemic: A machine learning approach. International Journal of Environmental Research and Public Health, 19(4), 2430. 10.3390/ijerph19042430 35206617 PMC8878508

